# Microstructural evaluation of the brain with advanced magnetic resonance imaging techniques in cases of electrical status epilepticus during sleep (ESES)

**DOI:** 10.55730/1300-0144.5754

**Published:** 2023-10-25

**Authors:** Hanife Gülden DÜZKALIR, Barış GENÇ, Safiye Güneş SAĞER, Ayberk TÜRKYILMAZ, Hediye Pınar GÜNBEY

**Affiliations:** 1 Department of Radiology, Kartal Dr. Lütfi Kırdar City Hospital, İstanbul, Turkiye; 2 Department of Radiology, Samsun Education and Research Hospital, Samsun, Turkiye; 3 Department of Pediatric Neurology, Kartal Dr. Lütfi Kırdar City Hospital, İstanbul, Turkiye; 4 Department of Medical Genetics, Faculty of Medicine, Karadeniz Technical University, Trabzon, Turkiye

**Keywords:** Electrical status epilepticus during sleep, microstructural analysis, morphometry, genetic mutation, tractus-based spatial statistics

## Abstract

**Background/aim:**

The cause and treatment of electrical status epilepticus during sleep (ESES), one of the epileptic encephalopathies of childhood, is unclear. The aim of this study was to evaluate possible microstructural abnormalities in the brain using advanced magnetic resonance imaging (MRI) techniques in ESES patients with and without genetic mutations.

**Materials and methods:**

This research comprised 12 ESES patients without structural thalamic lesions (6 with genetic abnormalities and 6 without) and 12 healthy children. Whole-exome sequencing was used for the genetic mutation analysis. Brain MRI data were evaluated using tractus-based spatial statistics, voxel-based morphometry, a local gyrification index, subcortical shape analysis, FreeSurfer volume, and cortical thickness. The data of the groups were compared.

**Results:**

The mean age in the control group was 9.05 ± 1.85 years, whereas that in the ESES group was 9.45 ± 2.72 years. Compared to the control group, the ESES patients showed higher mean thalamus diffusivity (p < 0.05). ESES patients with genetic mutations had lower axial diffusivity in the superior longitudinal fasciculus and gray matter volume in the entorhinal region, accumbens area, caudate, putamen, cerebral white matter, and outer cerebellar areas. The superior and middle temporal cortical thickness increased in the ESES patients.

**Conclusion:**

This study is important in terms of presenting the microstructural evaluation of the brain in ESES patients with advanced MRI analysis methods as well as comparing patients with and without genetic mutations. These findings may be associated with corticostriatal transmission, ictogenesis, epileptogenesis, neuropsychiatric symptoms, cognitive impairment, and cerebellar involvement in ESES. Expanded case-group studies may help to understand the physiology of the corticothalamic circuitry in its etiopathogenesis and develop secondary therapeutic targets for ESES.

## 1. Introduction

Electrical status epilepticus during sleep (ESES) is a type of epileptic encephalopathy that develops in childhood, worsens in middle and late childhood, and may heal spontaneously before puberty [1]. Cognitive impairment, electrical status epilepticus during sleep, and epileptiform activity without rapid eye movements are characteristic findings. The disease spectrum includes frontal lobe executive dysfunctions, behavioral abnormalities, learning problems, and intellectual deficiencies. Early detection and treatment are critical to prevent serious mental problems and resistant epilepsies [2,3].

Cranial magnetic resonance imaging (MRI) is the recommended imaging modality for ESES diagnosis. In the literature, ESES studies have mainly focused on the thalamus. Experimental studies have linked the amplification of oscillatory discharges in the cortico-thalamo-cortical (CTC) network to lesions in the reticular nucleus of the thalamus [3]. Thalamic volume loss, early developmental lesions, and functional impairment have been linked to CTC network disruption [4]. Although thalamic lesions have been linked to ESES [3], the cause of ESES without structural abnormalities is still not clearly understood.

Conventional MRI can detect macrostructural abnormalities such as thalamic damage in ESES, but microstructural changes in grey and white matter (WM) are difficult to detect with conventional sequences. One of the advanced MRI methods, diffusion tensor imaging (DTI), objectively measures water transport to characterize WM microstructure in vivo noninvasively. The main DTI parameters are fractional anisotropy (FA), which gives a summary measure of microstructural integrity; mean diffusion (MD), which describes overall diffusion and gives a measure of average diffusion in 3 directions; radial diffusion (RD); and axial diffusion (AD), which provides more explicit and specific information about the microstructure. Tractus-based spatial statistics (TBSS) is an automated, voxel-based statistical analytical DTI method developed to objectively compare diffusion properties between subjects independently of the observer [5]. Voxel-based morphometry (VBM) compares groups using a probability map based on the likelihood of gray matter (GM), WM, or cerebrospinal fluid (CSF) in voxels [6]. The local gyrification index measures the amount of folding, detects folding differences, and may indicate developmental disorders [7]. Subcortical shape analysis can precisely detect shape changes before volume changes [8].

The aim of this study was to evaluate possible microstructural abnormalities in the brain in ESES patients with and without genetic mutations using DTI TBSS, VBM, the local gyrification index, subcortical shape analysis, FreeSurfer volume, and cortical thickness analysis methods in cranial MRI.

## 2. Materials and methods

### 2.1. Study design

This observational cross-sectional study was performed at a single center. The study was performed in adherence with the Declaration of Helsinki,. The study was authorized by the ethics committee of our hospital. Written informed consent was given by the parents.

### 2.2. Patients and control groups

ESES patients (n = 20) aged 5–17 years were included in the study. The inclusion criteria were:

- A diagnosis of ESES by a pediatric neurologist based on the International League Against Epilepsy categorization, electrical status epilepticus during slow wave sleep and generalized bilateral symmetrical spike and slow wave discharges on electroencephalogram.
- The exclusion of other epileptic syndrome diagnoses.
- The absence of any other underlying etiological pathology (corpus callosum disruption or lesion, localized cortical lesion, hippocampal atrophy, cerebral or cerebellar atrophy, polymicrogyria or heterotopia, arachnoid cyst, hydrocephalus, perinatal ischemia, or thalamic lesion).

Of the patients, 4 were excluded due to thalamic asymmetry, 2 due to structural abnormalities, and 2 due to prenatal ischemia sequelae. Thus, 12 ESES patients were included.

The control group consisted of age- and sex-matched healthy patients who were referred to our radiology clinic for MRI scanning and had no abnormal findings on brain MRI, no clinical or laboratory data that could affect their neurological status, and no known diseases. Patients with MRI artifacts and incidental benign abnormalities such as arachnoid cysts and giant cisterna magna were excluded from the control group.

### 2.3. Molecular study

For the molecular study, 2 mL of peripheral blood was drawn from each ESES patient and into EDTA tubes. The QIAamp DNA Blood Mini QIAcube Kit (Qiagen, Hilden, Germany) was used to isolate genomic DNA from peripheral blood leucocytes according to the manufacturer’s instructions. All of the coding regions of the patients’ human genomes were sequenced using the QIAseq Human Exome Kit according to the manufacturer’s instructions (Qiagen). Paired-end sequencing (150 bp) was performed using a NovaSeq 6000 device (Illumina Inc., San Diego, CA, USA) according to the manufacturer’s instructions. The guardians of the patients were informed about the study in person, face-to-face, and written informed consent for the genetic analysis was obtained.

### 2.4. MRI technique and analysis methods

Imaging was performed with a 1.5 Tesla (T) MRI scanner (Philips Ingenia, Netherlands). Axial spin echo T1-weighted (repetition time/echo time (TR/TE) = 470–570/12–30 ms), axial and sagittal T2-weighted (TR/TE = 4500–6000/90–110 ms), axial and coronal fluid-attenuated inversion recovery (TR/TE = 6000–9000/100–120 ms), diffusion weighted imaging (DWI) (b = 0, 500, 1000), axial diffusion tensor imaging (DTI) (single-shot spin echo-planar imaging and acceleration factor 2 (TR/TE = 7721/96 ms, slice thickness = 2 mm, field of view (FOV) = 230, matrix = 128 × 128, number of excitations = 2, 2 mm, isotropic resolution, interslice gap = 0 mm, number of gradient directions = 16, b values = 0 and 1000 s/mm^2^), high-resolution 3D-T1W (TR/TE = 7. 2/33 ms, matrix = 256 × 256 pixels, number of signals averaged = 1, FOV = 256 mm, slice thickness = 1 mm; gap = 0 mm; flip angle = 8°) sequences were used to obtain structural images of the whole brain for anatomical reference. All of the sequences, except those 3D-T1W, were acquired with a slice thickness of 5 mm.

The images were evaluated by a radiologist experienced in neuroradiology. The ESES patients with and without genetic mutations and the control group were compared.

#### 2.4.1. DTI and TBSS analysis

**Figure 1:**
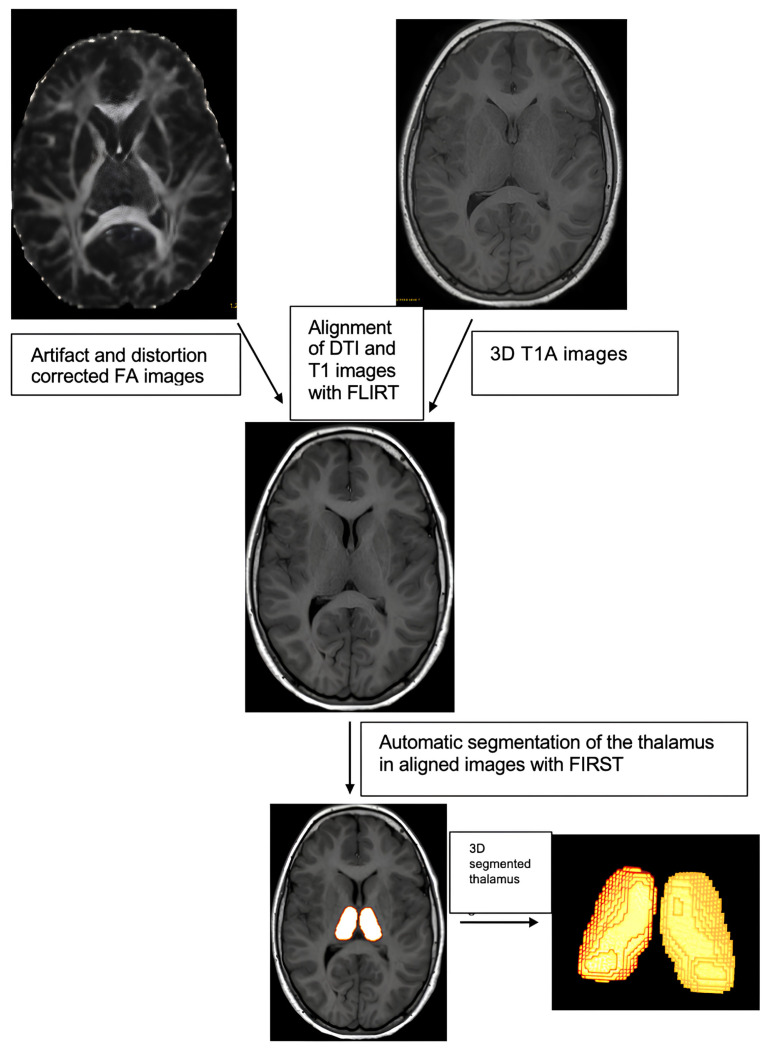
Schematic representation of the thalamus-specific analysis technique.

**Figure 2:**
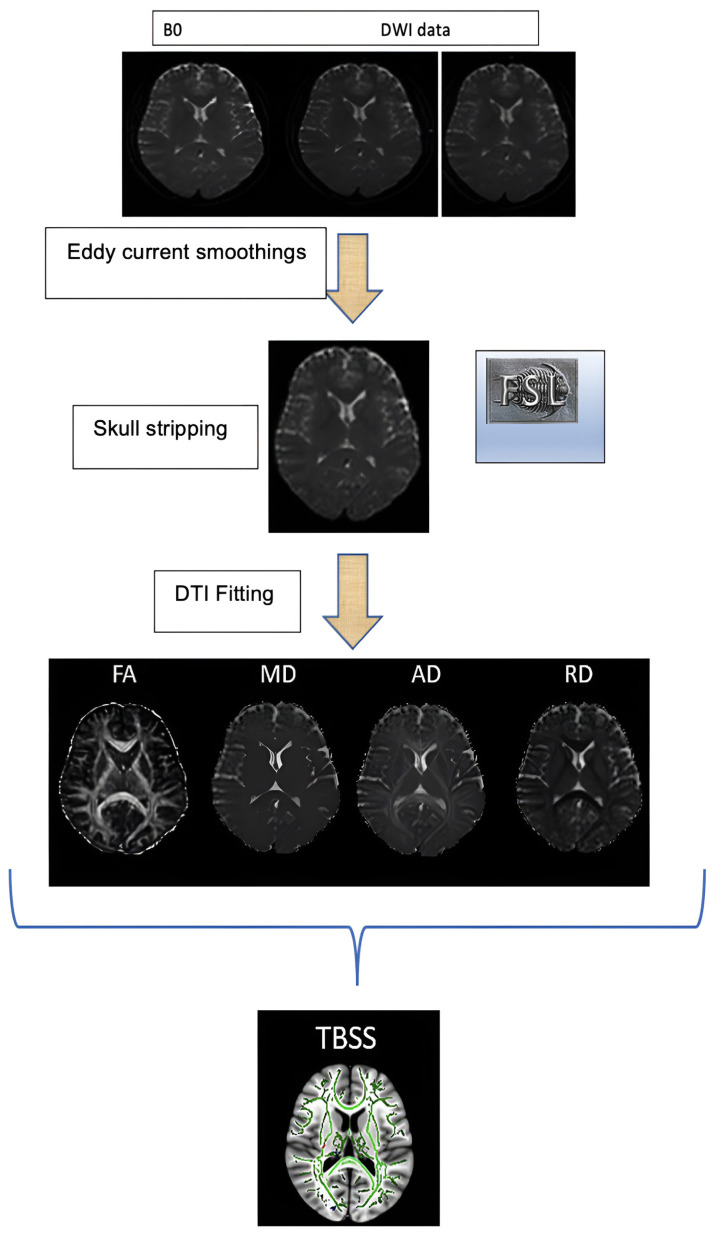
Schematic representation of the TBSS analysis.

The DTI and TBSS analysis began with DTI eddy current artifact adjustments on the cranial MR images. Then, FA, AD, MD, and RD maps were obtained with DTIFit. The Forensic Science Laboratory (FSL)-Oxford Centre for Functional Magnetic Resonance Imaging of the Brain’s (FMRIB) Integrated Registration and Segmentation Tool (FIRST), a model-based registration and segmentation program, automatically segmented the 3-dimensional (3D)-T1W thalamus images. The FMRIB Linear Image Registration Tool (FLIRT) program aligned the T1W and DTI images. The mean FA, MD, RD, and AD values of the segmented thalamus mask volumes of the aligned images were automatically calculated (Figure 1). The TBSS technique, which is part of the FSL software, was used for the DTI analysis of the WM tract microstructural changes (Figure 2). The Mann–Whitney U test was used to compare the thalamus FA, MD, RD, and AD values of the groups.

#### 2.4.2. VBM-local gyrification index analysis

The 3D T1W images were analyzed using the computational anatomy toolbox (CAT12) (http://dbm.neuro.uni-jena.de/cat/) program implemented in SPM12 (https://www.fil.ion.ucl.ac.uk/spm) running in MATLAB (version 2013a, MathWorks, Natick, MA, USA). The hemispheric surface was reconstructed via projection-based thickness (PBT) automatic reconstruction [9]. The GM, WM, and CSF were analyzed using the original T1W images. The GM images were normalized to an Institute of Neurology (MNI) template. The CAT12 was homogeneity-checked [6]. The GM, WM, and CSF volumes were summed. The GM volume was compared across groups, and voxel density analysis was performed. The indentation index is the ratio of absolute mean curvature of the outer brain surface to the outer surface excluding sulci [10]. Using a standard thalamic mask, the whole brain and 2 regions of interest (ROIs) were compared. All of the ROIs were analyzed simultaneously for the left and right hemispheres, and the automatic anatomical labeling (AAL) atlas revealed the affected brain regions [6].

#### 2.4.3. Subcortical shape analysis

This method used FSL-FIRST to segment the T1W images. The deformable surfaces of the deep GM structures automatically parameterized the volumetric labels. Multivariate Gaussian assumptions collected and modeled the normalized surface intensities. Next, the shape was averaged using the main components. After automated segmentation, all of the subcortical structures were carefully confirmed. Multivariate testing in the 3D vertex coordinates was performed in the vertex-wise shape analysis. The false discovery rate (FDR) or surface-based cluster corrections were evaluated for each vertex separately. The coordinates of each vertex were transferred back and studied in standard space or another space. Alignment eliminated the global rotation and translation between the subjects. For each vertex, the multivariate general linear model (MVGLM) with Pillai’s Trace provided a multivariate F-test sensitive to coordinate changes. The variables were age, sex, and total brain volume. The thalamus, hippocampus volumes, amygdala, caudate, and putamen were extracted. Statistical significance was accepted as p < 0.05 (FDR-corrected) [11]. IBM SPSS Statistics for Windows 22.0 (IBM Corp., Armonk, NY, USA) was used for the statistical comparison.

#### 2.4.4. FreeSurfer volume and cortical thickness analysis

For accuracy, surface-based cortical thickness analysis models were made of the whole surface. FreeSurfer analysis is the gold standard for cortical thickness measurements, which employs the surface-based technique, and takes a long time to analyze [12]. FreeSurfer (http://surfer.nmr.mgh.harvard.edu/) with the recon-all process flow was used to build a 3D cortical surface model from the T1W images [13]. After an automated Talairach transformation, nonbrain tissue removal, hemisphere separation, and cerebellum and brain stem removal were performed. Then, the GM and WM surface borders were reconstructed [12]. Cortical thickness was accepted as the pial surface-WM distance [13]. After the automatic reconstruction, possible misclassification was visually checked. Using the Desikan–Killiany atlas, the mean cortical thickness values were averaged for 34 ROIs [14]. Then, the ROI results of both groups were statistically compared.

### 2.5. Statistical analysis

SPSS was used to perform the statistical analysis. Complementary statistical methods were used to evaluate the data (mean, standard deviation, median, frequency, percentage, minimum, and maximum). Quantitative variables without normal distribution were compared between 2 groups using the Mann–Whitney U test and Kruskal–Wallis test. Patient and healthy group comparisons of the nonnormal variables was performed using the Wilcoxon signed-rank test. Statistical significance was accepted as p < 0.05. In the VBM analysis, a 1-tailed significance threshold was set at voxel-level uncorrected p < 0.001 and cluster-level family-wise error (FWE)-corrected p < 0.05 [15].

## 3. Results

Herein, 20 ESES patients were initially considered. After taking into account the exclusion criteria, 12 ESES patients (33.3% female and 66.7% male) and 12 healthy controls (33.3% female and 66.7% male) were compared. The age and sex distribution was not significantly different between the groups. Whole exome sequencing (WES) was used to examine the ESES patients for genetic mutations. In 8 patients, one or more epilepsy genes, *GRIN2A, TET3, SCN1A, SCN8A, ADGRV1, DLG4*, and *SLC12A5*, were found, and 12 patients had no genetic mutations. Based on the exclusion criteria, 6 ESES patients with genetic mutations and 6 without were included.

**Figure 3:**
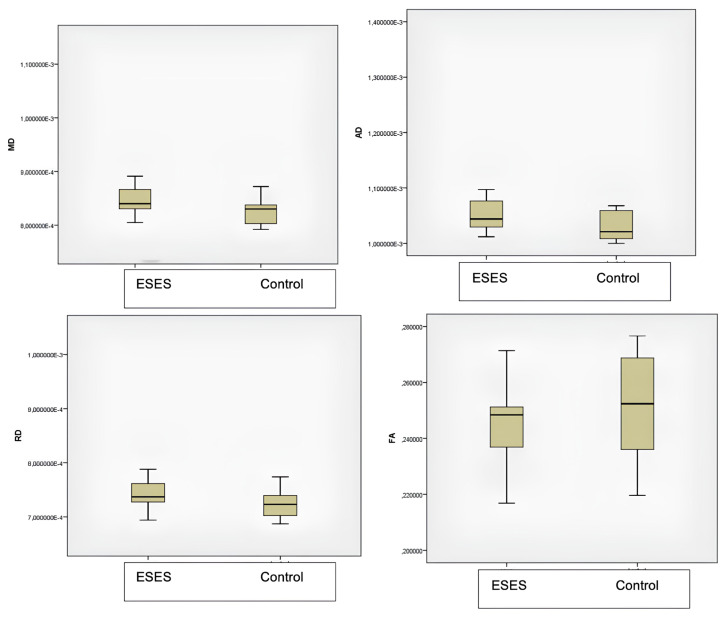
ROI analysis of the AD, FA, MD, RD values in the ESES and control groups.

**Figure 4:**
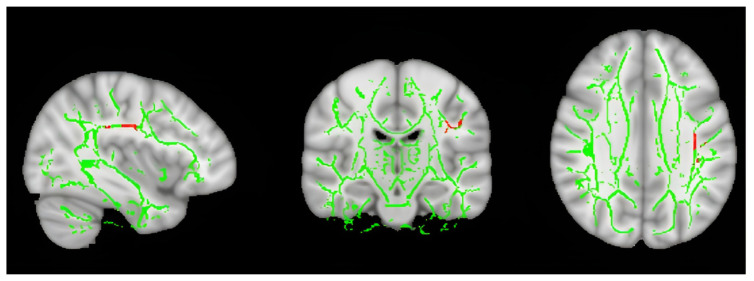
Comparison of the ESES groups with and without genetic mutations, areas showing increased AD in patients with mutations.

Figure 3 shows the thalamus-specific volume-of-interest (TBSS-VOI) analysis findings from the DTI and TBSS analysis. The ESES patients had significantly higher MD in their thalamus than the controls (p = 0.042). Differences between the groups in the FA, AD, and RD were not significant (p > 0.05). The WM TBSS analysis showed no significant differences in the FA, MD, AD, and RD between the control, mutation-positive, and mutation-negative groups (p > 0.05) (Table 1). The Kruskal–Wallis test showed that the mutation-positive ESES patients had decreased AD in the superior longitudinal fasciculus (SLF) (Figure 4).

**Figure 5:**
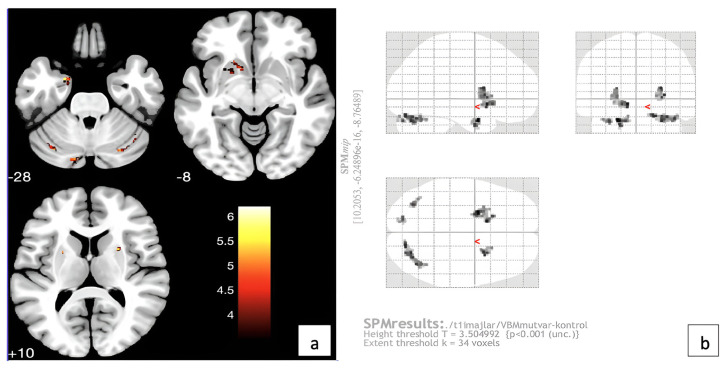
VBM analysis: (a) Locations of decreased GM volume in the mutation-positive ESES: Left entorhinal area, left putamen, left cerebral WM, left accumbens area, left caudate, right cerebellum exterior, right putamen, right cerebral WM, left cerebellum exterior, left cerebellum exterior, and (b) the appearance of the same areas in the glass brain image.

VBM analysis, which analyzes the chance of a voxel having GM-WM-BOS, showed no statistically significant difference between the mutation-negative and control groups. Mutation-positive patients showed a decreased GM volume in the left entorhinal area, bilateral putamen, bilateral cerebral WM, left accumbens, left caudate, and bilateral cerebellum exterior (Table 2, Figure 5a). Figure 5b shows glass brain images of the same locations.

Specific curvature differences were analyzed in the ESES patients with genetic abnormalities using a local gyrification index analysis since it may indicate developmental disorders. No significant difference was found across the groups.

Subcortical shape analysis was used to extract and statistically compare 15 right and left subcortical structures and the 3D thalamus shape. Shape changes precede the volume changes, which is what makes this method sensitive. The bilateral thalamus, accumbens, hippocampus, putamen, caudate nucleus, and amygdala of the patients were not significantly different.

**Figure 6:**
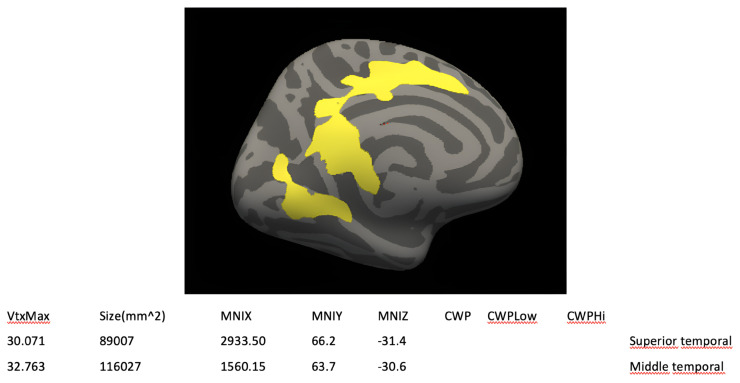
FreeSurfer volume and cortical thickness analysis results: areas with increased cortical thickness in the ESES group; superior temporal and middle temporal.

Figure 6 shows the FreeSurfer volume and cortical thickness analyses. The superior and middle temporal cortical thickness in the ESES patients were determined to increase using FreeSurfer.

## 4. Discussion

This microstructural brain study of ESES patients with and without genetic mutations utilizing DTI-TBSS analysis, VBM, a local gyrification index, subcortical shape analysis, and FreeSurfer volume-cortical thickness analysis differs from the literature. Multiple analytic approaches including thalamus-specific analysis assessed GM and WM structural changes in ESES patients.

In our TBSS-VOI analysis, ESES patients’ thalamus MD increased. The thalamus and thalamocortical circuit have been studied for ESES’s etiopathogenesis. The literature attributes thalamic physiological oscillations to the cyclic interaction between glutaminergic excitatory thalamocortical neurons in the dorsal thalamic nuclei and inhibitory GABAergic neurons in the reticular nuclei [16,17]. ESES patients exhibit thalamic involvement during interictal epileptic discharges [18], and early thalamic lesions may damage the corticothalamic circuit, causing ESES [19]. Parenchymal damage is usually present [20]. Thalamic volume and metabolic changes can be seen in ESES but are complex [1,21–23]. These studies cannot explain etiopathogenesis without structural thalamic lesions.

In our study, MD, which measures total diffusion, increased in ESES patients without thalamic lesions, indicating thalamic integrity loss and microstructural involvement. In the Cuprizone mouse model, AD (water molecule diffusion parallel to WM tracts) decreases, indicating axonal degeneration and early demyelination [24]. Genetically mutant ESES patients have decreased SLF AD. SLF is a WM structure with long, bidirectional projections between the prefrontal, temporal, occipital, and parietal cortices. The Human Connectome Project DTI template clarifies its anatomical characteristics but not its neurobehavioral functions. Its anatomical location suggests it contributes to several cognitive domains, including visuospatial non-verbal cognition and verbal memory. SLF DTI showed significant correlations between the left SLF, perceptual organization, and working memory [25]. In a pediatric study that evaluated the SLF’s microstructural integrity, neuropsychiatric functions, and cognitive domain, FA and AD levels were positively correlated with executive functions [26]. One pathophysiologic reason for cognitive decline, memory impairment, and attention deficit in our ESES patients may be decreased AD in the left SLF.

In genetically mutated ESES patients, VBM showed reduce GM volumes in the corpus striatum, accumbens, entorhinal, and cerebellar outer part. This study showed that seizures develop with spike-wave (SWD) discharges initiated by decreased cortical excitatory transmission to the striatum, decreased excitatory transmission from the neocortex to striatal fast-spiking interneurons, SWDs are suppressed when AMPA receptors in the striatum are potentiated, and disruption of cortico-striatal excitatory transmission in haplodeficient mice causes epilepsy [27]. Corticostriatal transmission may explain the corpus striatum GM volume reduction in our genetically mutated ESES patients. Large genetic-based studies on this field may help evaluate new disease treatment targets like DBS and develop treatments.

The mesolimbic pathway involving the nucleus accumbens (Nacc) stores memories and is a reward circuit [28]. Nacc abnormalities, Nacc neuron degeneration, and abnormal neuroactive substance distribution are linked to refractory epilepsy’s pathophysiology. In refractory epilepsy, Nacc involvement has been linked to motivational-emotional processes, limbic-motor interfaces, and clinical findings. It may be a therapeutic target [29]. The role of Nacc in ESES ictogenesis and epileptogenesis has been supported by decreased ventral striatum accumbens GM volume in our study. Our findings may explain ESES neuropsychiatric symptoms. However, they cannot explain the genetic mutation link, hence complete genetic studies are needed.

The entorhinal area transmits most cortical information to the hippocampus [30]. VBM study showed decreased entorhinal area GM volume in our genetically mutated ESES patients. Similarly, morphometric studies of mesial temporal epilepsy patients’ hippocampus-related regions indicate a volume decrease. ESES patients’ cognitive decline may be caused by entorhinal GM volume loss, which affects memory and memory-related paradigms [31].

Neuromodulation is being targeted beyond the epileptogenic center in epilepsy treatment. The cerebellum may be a therapeutic target in this scenario, and cerebellar modulation may strongly reduce seizures [32]. Cerebellar functional, structural, volumetric, and perfusional changes have been linked to epilepsy [33–36]. In addition to motor functions, the cerebellum is engaged in higher-order cognitive processes such hippocampus contextual computations [37, 38]. The numerous feed-back loops formed by input from the cortex via the pontine nuclei and output via the thalamus make the lateral cerebellar hemispheres part of the cerebrocerebellum [39]. Seizure susceptibility has been linked to the cerebellum’s role in brain state modulation, particularly sleep-wake cycle management [40–43]. Cerebellar GM loss preceded SUDEP in a recent research. The cerebellum has been connected to seizure networks aside from epilepsy [32]. Structural changes affect cognition and patient outcomes and must be studied. We found GM volume decrease in the cerebellum exterior in ESES patients, suggesting treatment targets.

Our study identified no causal association between ESES-emphasized CTC cycle damage and rearrangements and their consequences. Our limited DTI array, population’s lack of structural thalamic lesions, small sample size, and lack of longitudinal assessments may explain this. Longitudinal studies will be beneficial for tracking the progression of processes.

The local gyrification index and subcortical shape analyses did not differ for the thalamus, hippocampus, amygdala, caudate, and putamen. This may have been due to the fact that the cross-sectional model, sample size, sample characteristics, underlying genetic factors or heterogeneity, the fact that most patients had started drug treatment, the duration of the disease and seizures, and the variability of treatment duration were technically determined.

The superior and middle temporal cortical thickness increased in the FreeSurfer analysis. Cortical thickness changes have numerous causes. Contrary to the results herein, temporal cortical thickness decreases have been associated with epilepsy duration and seizure frequency in general [5,44,45]. In temporal lobe epilepsy, glial, neuronal, and synaptic rearrangements may cause neocortex neuronal structural abnormalities similar to those in the hippocampus [46,47]. Cortical thickness changes may indicate long-term functional reorganization [48,49]. In the current study, the temporal cortical thickness may have varied due to increased CTC cycle activation and reorganization in ESES, treatment start date, seizure type, duration of the ictal-postictal interval, and seizure frequency. Cortical changes affect seizure frequency, duration, severity, and physiology [50]. ESES cortical interactions and processes require large and varied investigations.

Antiepileptic drugs might prevent cognitive deterioration and promote neurodevelopment if sophisticated MRI technologies identified people at high risk of ESES or in the prodromal phase when seizures began [4]. To rule out seizure types that may cause focal thalamic volume reduction, interictal epileptiform discharges, and chronic exposure to antiepileptic drug treatment, its effects, and disease duration, a prospective study with a large group of patients is needed.

This study did have some limitations. The patients included those with and without genetic abnormalities, even though the majority had begun therapy. The small sample size, limited DTI array, and lack of literature on ESES cases with genetic alterations were other limitations. Further studies with untreated patients may help us to understand the pathogenesis of ESES.

In conclusion, increased MD without thalamic structural lesions in ESES patients suggested volume-independent microstructural damage. Cognitive decline may be linked to decreased AD in SLF in ESES patients with genetic abnormalities in TBSS studies. In the VBM analysis, GM volume decreases in the corpus striatum, accumbens area, entorhinal area, and cerebellum exterior in the ESES patients with genetic mutations in the current study may have been related to corticostriatal transmission, ictogenesis, epileptogenesis, neuropsychiatric symptoms, cognitive decline, and cerebellar involvement, and these locations may be therapeutic targets in comprehensive studies. In the FreeSurfer analysis, increased cortical thickness at the superior and middle temporal levels may be linked to thalamocortical circuit activation, patient restructuring or treatment, seizure types, duration of the ictal-postictal period, and seizure frequency. In ESES, studies that combine DTI, volumetric assessment, metabolic changes, and fMRI may help to explain the physiology and etiopathogenesis of the CTC cycle and develop new therapeutic targets.

## Figures and Tables

**Table 1:** Thalamus-specific tract-based spatial statistics (TBSS)-volume of interest (VOI) analysis results of the ESES patients compared to the healthy control group.

Group		Mean	SD^b^	p-value^c^
FA^a^	ESES	0.24318375	0.005431528	0.242
	Control	0.25105600	0.006151309	
MD^a^	ESES	0.00086583	0.000024712	0.042
	Control	0.00082491	0.000007601	
AD^a^	ESES	0.00107000	0.000081912	0.060
	Control	0.00103009	0.000027271	
RD^a^	ESES	0.00076367	0.000025483	0.124
	Control	0.00072245	0.000008137	

a: FA: fractional anisotropy, MD: mean diffusion, AD: axial diffusion, RD: radial diffusion,

b: SD: standard deviation,

c: Mann–Whitney U test.

**Table 2:** Decrease in GM volume observed in the genetic mutation-positive ESES patients compered to mutation-negative patients.

Cluster index	Labels^a^	Cluster size	x, y, z (mm)^b^	p-value^c^
1	Right cerebellum exterior 92%	78	8, −82, −32	6.08
2	Right cerebellum exterior 98%	245	33, −72, −24	5.72
	Right occipital fusiform gyrus 2%			
3	Left cerebellum exterior 100%	78	−33, −72, −28	5.47
4	Right cerebellum exterior 100%	68	24, −68, −21	5.16
5	Left entorhinal area 96%	80	−22, 0, −28	5.14
	Left fusiform gyrus 4%			
6	Left cerebellum exterior 94%	35	−27, −72, −24	5.09
	Left occipital fusiform gyrus 6%			
7	Left cerebellum exterior 100%	46	−39, −81, −28	4.60
8	Left putamen 100%	92	−24, 4, 10	4.58

a: Labels according to a Desikan Killiany atlas in CAT12. The percentiles given next to the regions show how much of the vertices in that cluster belong to that region,

b: According to the MNI coordinate system,

c: VBM at a threshold of voxel-level uncorrected p < 0.001 and cluster-level FWE corrected p < 0.05 (Poline et al., 1997) for omissions and superordinate errors.
